# Transient adrenal insufficiency in diffuse large B cell lymphoma patients after chemotherapy with short-course, high-dose corticosteroids

**DOI:** 10.1007/s00277-018-3470-y

**Published:** 2018-08-14

**Authors:** Weerapat Owattanapanich, Sirinart Sirinvaravong, Kittima Suphadirekkul, Taweesak Wannachalee

**Affiliations:** 1 Division of Hematology, Department of Medicine, Faculty of Medicine Siriraj Hospital, Mahidol University, 2 Wanglang Road, Bangkoknoi, Bangkok, 10700 Thailand; 2 Division of Endocrinology and Metabolism, Department of Medicine, Faculty of Medicine Siriraj Hospital, Mahidol University, Bangkok, Thailand

**Keywords:** Diffuse large B cell lymphoma, Adrenal insufficiency, R-CHOP regimen

## Abstract

Data on the rate of adrenal insufficiency (AI) in patients receiving short-course and high-dose corticosteroids are limited. In this study, we aimed to determine the incidence of AI in newly diagnosed, diffuse large B cell lymphoma (DLBCL) patients after receiving rituximab, cyclophosphamide, doxorubicin, vincristine, and prednisone [or prednisolone] (R-CHOP/CHOP) regimen. We enrolled newly diagnosed DLBCL patients who were scheduled to receive 6–8 cycles of R-CHOP/CHOP regimen. One-microgram adrenocorticotropic hormone (ACTH) stimulation tests were performed at the study entry and 3 weeks after each cycle of chemotherapy (CMT). AI was defined by a peak-stimulated serum cortisol of less than 18 μg/dL. For patients who had AI after completing a course of CMT, 1-μg ACTH stimulation tests were carried out at 60 and 90 days after the last CMT cycle to assess the duration of hypothalamic-pituitary-adrenal (HPA) axis recovery. Ten DLBCL patients were included in this study, with a total of 84 1-μg ACTH stimulation tests. Their mean age was 52 years. AI occurred in 3 out of the 10 patients (30%). The first occurrence of AI was after the third CMT cycle, and the incidence was highest after the fifth cycle. Adrenal function recovered completely 3 to 5 weeks after completing the course of CMT, except for 1 patient, whose HPA axis suppression persisted 90 days after the last CMT cycle. Receiver operating characteristic (ROC) analysis revealed that a basal cortisol level of < 8.7 μg/dL was predictive of AI, with a sensitivity and specificity of 80% and 72.2%, respectively. Transient HPA axis suppression can occur in DLBCL patients receiving R-CHOP/CHOP regimen. We strongly encourage careful observation and examination for potential adrenal insufficiency in such patients, particularly after the fifth cycle of chemotherapy.

## Introduction

Secondary adrenal insufficiency (AI) is an adrenal hypofunction caused by inadequate levels of adrenocorticotropic hormone (ACTH) being produced by the pituitary gland. The common symptoms of AI are fatigue, weakness, weight loss, nausea, and vomiting. The most common cause of secondary AI is chronic exogenous steroid use, and it has been observed to have diverse routes of administration, including systemic, topical, intra-articular, inhaled, and ocular [1–5]. The occurrence and duration of the hypothalamic-pituitary-adrenal axis (HPA) suppression depend on the duration and dosage of the exogenous steroids. However, solid evidence for the rate of adrenal insufficiency in patients receiving multiple rounds of short-term and high-dose corticosteroids is scarce. Data on the duration of the HPA axis suppression are also lacking.

Diffuse large B cell lymphoma (DLBCL) is classified as the most common lymphoma subtype both in Thailand and worldwide. An R-CHOP (rituximab, cyclophosphamide, doxorubicin, vincristine, and prednisone [or prednisolone]) regimen remains the standard protocol for DLBCL treatment. Each cycle consists of 5 consecutive days of high-dose prednisolone [6]. However, the incidence of AI arising from this regimen has not been evaluated.

According to a previous study of patients with acute exacerbation of chronic obstructive pulmonary disease (AECOPD) who received a 14-day course of systemic corticosteroids (1 dose of 40 mg of methylprednisolone, followed by 13 days of 40 mg of prednisolone per day), HPA axis suppression occurred in up to 89% of patients after 14 days of treatment, and it remained in 33% of patients after 3 weeks of glucocorticoid withdrawal [2]. These data indicate that transient acute adrenal insufficiency can occur among patients who receive only a short course and supraphysiologic doses of systemic corticosteroids, and prolonged HPA axis suppression is also possible. Other studies of acute lymphoblastic leukemia (ALL) patients who received high-dose corticosteroids during the induction phase of chemotherapy reported an incidence of AI ranging from 20 to 100% [3]. The duration of the HPA axis suppression also varied from weeks to months in those studies.

Extended suppression of the HPA axis can lead to an adrenal crisis, especially if it is triggered by an acute illness such as by dehydration, by surgery, or by an infection which commonly occurs in patients with a hematologic malignancy who have been receiving chemotherapy [7–10]. This presents a very challenging clinical situation as symptoms of the primary disease as well as any complications (such as sepsis or febrile neutropenia) could mimic the clinical manifestations of adrenal insufficiency, leading to under-recognition of adrenal insufficiency in this patient group. Furthermore, since the clinical manifestations overlap, physicians encounter difficulties determining whether it is AI or the primary disease that is contributing to such symptoms.

We conducted this study to determine the incidence of AI in newly diagnosed DLBCL patients after receiving high-dose steroid treatment, and to evaluate the duration of the HPA axis recovery.

## Design and methods

### Patients and procedures

This study was conducted among 17 patients aged more than 18 years who received an R-CHOP/CHOP regimen consisting of pulse high-dose steroid therapy for newly diagnosed DLBCL at Siriraj Hospital between March 2, 2016 and January 16, 2017. Informed written consent was obtained from every patient. All patients received between 6 and 8 cycles of an R-CHOP/CHOP regimen, which comprised 100 mg/day of prednisolone for 5 continuous days in each cycle. Patients with a prior history of AI, adrenal masses on imaging studies, or a previous exposure to corticosteroid treatment or a steroid-compounded medicine were excluded. Our study was approved by the Siriraj Institutional Review Board (SIRB) and followed the guidelines outlined in the Declaration of Helsinki and all of its subsequent amendments. This study was supported by the Routine to Research Unit, Siriraj Hospital.

The 1-μg (low-dose) ACTH stimulation test is considered to be the method to evaluate the short-term effect of HPA axis suppression by exogenous corticosteroids and can detect a more subtly impaired adrenal function. The test was initiated between 8.00 and 9.00 am. ACTH was freshly prepared from 250 μg Synacthen diluted with normal saline to 1 μg/mL. Blood samples for serum total cortisol, serum albumin, and plasma ACTH were drawn simultaneously before testing. One milliliter of diluted, synthetic ACTH was administered intravenously in each subject, and blood samples for cortisol were collected at baseline, and 20, 30, 40, and 60 min after ACTH administration.

With our patients, the 1-μg ACTH stimulation test was performed at baseline (before initiating chemotherapy) and 3 weeks after every cycle of chemotherapy. In this setting, AI was defined as the peak-activated serum cortisol of less than 18 μg/dL. Patients who had a peak-stimulated cortisol of < 18 μg/dL at the study entry were excluded [11]. Patients who had inadequate adrenal response (i.e., a peak stimulated cortisol of < 18μg/dL) after the last cycle of chemotherapy underwent further 1-μg ACTH stimulation tests at another 30 and 60 days to determine the timing of the HPA axis recovery.

Clinical manifestations, including symptoms and physical signs of all patients, were collected throughout the study period. Computed tomography of the adrenal gland was performed after the fourth and the last chemotherapy cycles to detect new adrenal lesions or metastasis.

### Laboratory assays

A venous catheter was inserted in each subject 30 min before the test to avoid stress from the venipuncture. Six milliliters of blood was collected from the catheter, and its serum was analyzed by commercial assay. The serum total cortisol levels were measured using the methodology of the electrochemiluminescence immunoassay (ECLIA) competition principle. The normal reference range for the 8 a.m. cortisol level was 3–18 μg/dL. The intra-assay and inter-assay coefficients of variation (CVs) of this test were 1–1.8% and 1.43–3.25%, respectively. The plasma ACTH levels were measured by immunoradiometric assay (IRMA) in the Endocrine Laboratory Unit of Siriraj Hospital. The normal reference range for the 8 a.m. ACTH level was 10–65 pg/mL. The intra-assay and inter-assay CVs of this test were 2.9% and 4.8%, respectively. Serum albumin was measured by ECLIA on the same day (normal reference range, 3.5–5.2 g/dL).

### Statistical analysis

The clinical characteristics of each group were presented as the median and interquartile range and standard deviation or percentage. Continuous data were analyzed by the paired *t* test or the Mann-Whitney *U* test. Categorical data for comparisons between the groups were analyzed by the chi-squared test or Fisher’s exact test. Parametric and nonparametric analyses were analyzed using IBM SPSS Statistics for Windows, version 24.0 (IBM Corp., Armonk, NY, USA), and a *p* value of < 0.05 was considered as statistically significant.

## Results

### Baseline characteristics

**Fig. 1:**
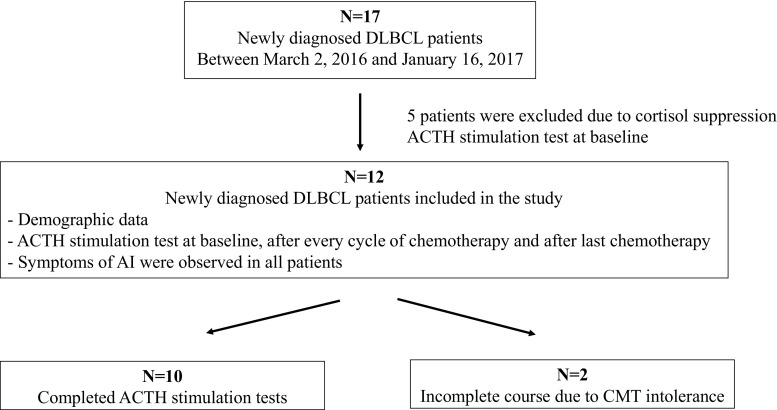
Flow chart of patient recruitment

Seventeen patients with newly diagnosed DLBCL, who were scheduled to receive an R-CHOP/CHOP regimen for 6–8 cycles, were enrolled in this study between March 2016 and January 2017. Five patients who had a peak stimulated cortisol of < 18 μg/dL after the 1-μg ACTH stimulation test at the study entry were excluded. Two patients were excluded due to an intolerance to chemotherapy. Therefore, 10 patients completed this study (Fig. 1). Their mean age was 52.2 years (S.D., 8 years), with a male predominance. Four patients had comorbidities; of those, 2 (20% of the study population) had type 2 diabetes mellitus, 2 (20%) had hypertension, and 1 (10%) had coronary artery disease. Three fifths (6) of the patients had an advanced stage, according to Ann Arbor Staging, and extranodal involvement. None of the patients had an ECOG score of more than 2 points. Eighty percent (8) of the patients received an R-CHOP regimen, while the remaining 20% (2) were treated with a CHOP regimen (Table 1).

**Table 1:** Baseline characteristics of 10 DLBCL patients

Clinical characteristics	*N* (%)
Age (years), mean ± standard deviation	52.2 ± 8
Sex	
Male	6 (60%)
Female	4 (40%)
Weight (kg), mean ± standard deviation	69.2 ± 20.1
Height (cm), mean ± standard deviation	164.7 ± 7.2
Comorbidities	
Diabetic mellitus	2 (20%)
Hypertension	2 (20%)
Dyslipidemia	1 (10%)
Coronary artery disease	1 (10%)
Chronic kidney disease	1 (10%)
Chronic hepatitis B	1 (10%)
Chronic hepatitis C	1 (10%)
Hemophilia	1 (10%)
Performance status*	
0	5 (50%)
1	4 (40%)
2	1 (10%)
Ann Arbor Stage	
1	2 (20%)
2	2 (20%)
3	1 (10%)
4	5 (50%)
B symptoms	
Absent	8 (80%)
Present	2 (20%)
Bone marrow involvement	
Absent	8 (80%)
Present	2 (20%)
No. of extranodal sites	
0	4 (40%)
1	4 (40%)
2	1 (10%)
3	1 (10%)
Bulky	
Absent	9 (90%)
Present	1 (10%)
Standard International Prognostic Index score (IPI-score)	
0	4 (40%)
1	–
2	2 (20%)
3	4 (40%)
4–5	–
Chemotherapy	
R-CHOP	8 (80%)
CHOP	2 (20%)
Baseline serum cortisol (μg/dL), mean ± standard deviation	11.1 ± 3.0
Baseline ACTH level (pg/mL), mean ± standard deviation	41.3 ± 24.8

*IPI* Standard International Prognostic Index score, *LN* lymphadenopathy, *ACTH* adrenocorticotropic hormone

*Performance status was defined according to the criteria of the Eastern Clinical Oncology Group (with an increasing score indicating declining performance)

### Cortisol and clinical evaluation

**Fig. 2:**
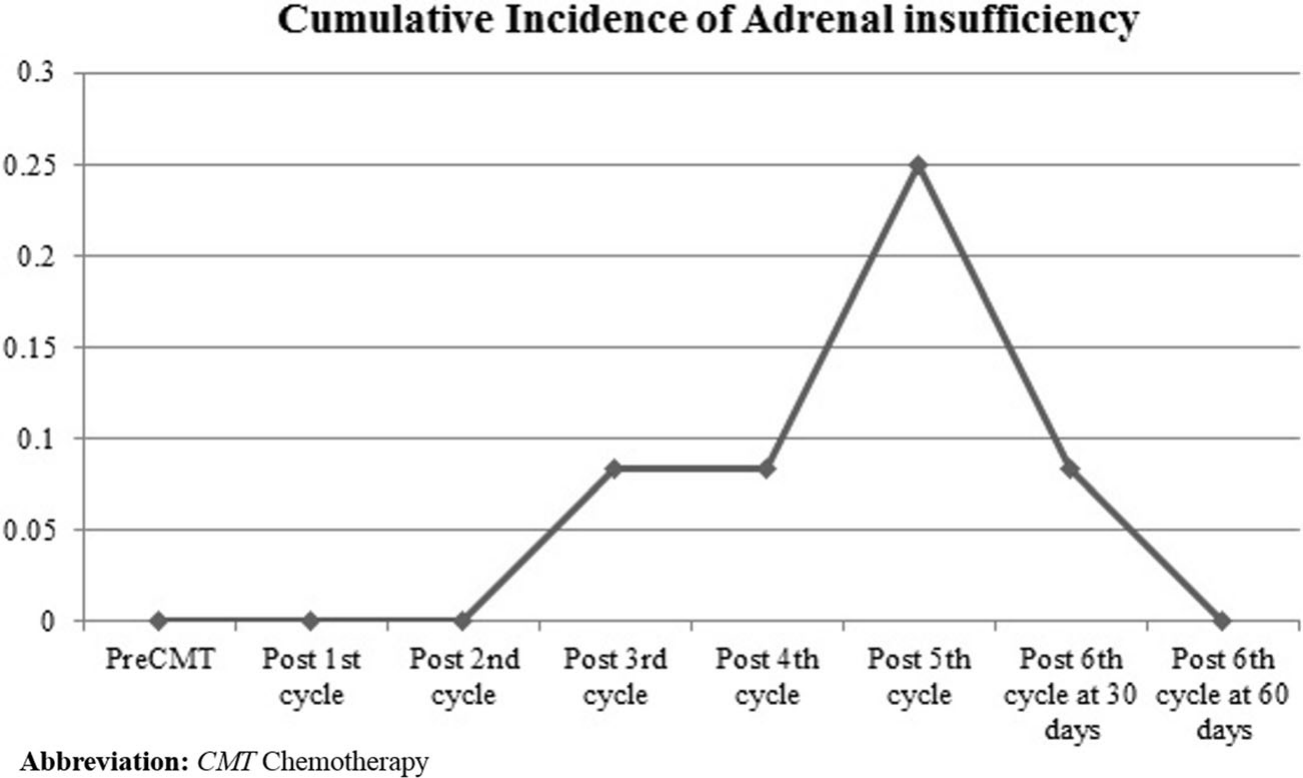
Cumulative incidence of adrenal insufficiency

**Fig. 3:**
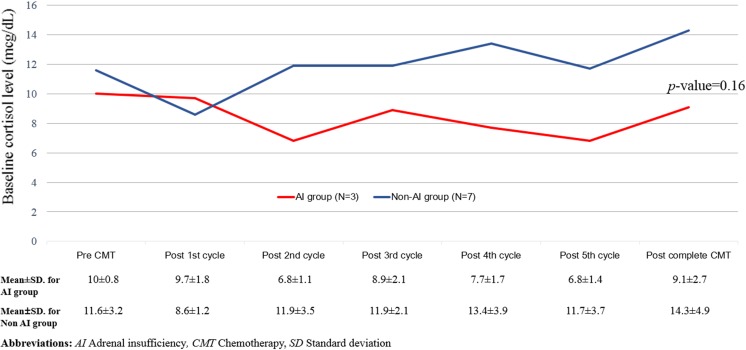
Basal cortisol levels of AI and non-AI groups

At diagnosis, the baseline serum morning cortisol levels of all patients were within the normal range, with a mean level of 11.1 μg/dL (S.D., 3.0 μg/dL). The serum baseline albumin levels were > 3.5 g/dL for all patients and remained at > 3 g/dL during the course of the chemotherapy. A total of 84 1-μg ACTH stimulation tests were performed. AI occurred in 3 out of the 10 patients. We found that the first occurrence of AI was after the third chemotherapy cycle, and the highest incidence was after the fifth cycle (*p* = 0.008; Fig. 2). We observed a trend of lower basal cortisol levels in patients who developed AI than those in the non-AI group, although the difference was not statistically significant (*p =* 0.16; Fig. 3). It is worthwhile noting that none of the patients had febrile neutropenia or received medications that could alter steroid metabolism.

The patients who had AI were significantly younger than those who did not. The mean ages of the AI and non-AI groups were 34.7 and 64.1 years, respectively (*p* = 0.014, 95% CI 2.52–47.56). Likewise, the AI group members were more likely to be in an early stage of disease (stages I–II) compared to the non-AI group (*p* = 0.03; Table 2). Therefore, a younger age and an early disease stage appeared to be predictors of AI after chemotherapy. The occurrence of AI was irrelevant to comorbidities, performance status, or advanced disease status. Interestingly, none of the patients who had abnormal adrenal testing experienced symptoms of AI, such as hypotension, weakness, and tiredness. They neither developed erectile dysfunction nor reported decline in sexual function after R-CHOP regimen.

**Table 2:** Clinical-characteristics comparison of AI group (*N* = 3) and non-AI group (*N* = 7)

Characteristics	Adrenal insufficiency		OR (95% CI)	*p* value
	Present	Absent		
Mean age (years)	34.7	64.1	9.78 (2.52–47.56)	*0.03*
Sex				
Male Female	2 1	4 3	0.76 (0.03–11.28)	1.00
Comorbidities			N/A	0.17
Present Absent	0 3	5 2		
Performance status			N/A	0.25
0 1 2	3 0 0	2 4 1		
Ann Arbor stage			N/A	*0.03*
Early (1.2) Advanced (3.4)	3 0	1 6		
B symptoms			N/A	1.0
Present Absent	0 3	2 5		
Bone marrow involvement			N/A	1.0
Present Absent	0 3	2 5		
No. of extranodal sites			N/A	1.0
0 1 2 3	2 1 0 0	2 3 1 1		
Bulky			N/A	1.0
Present Absent	0 3	1 6		
Generalized LN			N/A	0.2
Present Absent	0 3	4 3		
Localized LN			2.66 (0.15–45.14)	1.0
Present Absent	2 1	3 4		
IPI score			N/A	0.13
0 2 3	3 0 0	1 2 4		
Chemotherapy			3 (0.12–73.64)	1.0
R-CHOP CHOP	2 1	6 1		
Basal cortisol (μg/dL)	10.0	11.6	2.1 (− 3.3–6.38)	0.49

*IPI* Standard International Prognostic Index score, *LN* lymphadenopathy

**Fig. 4:**
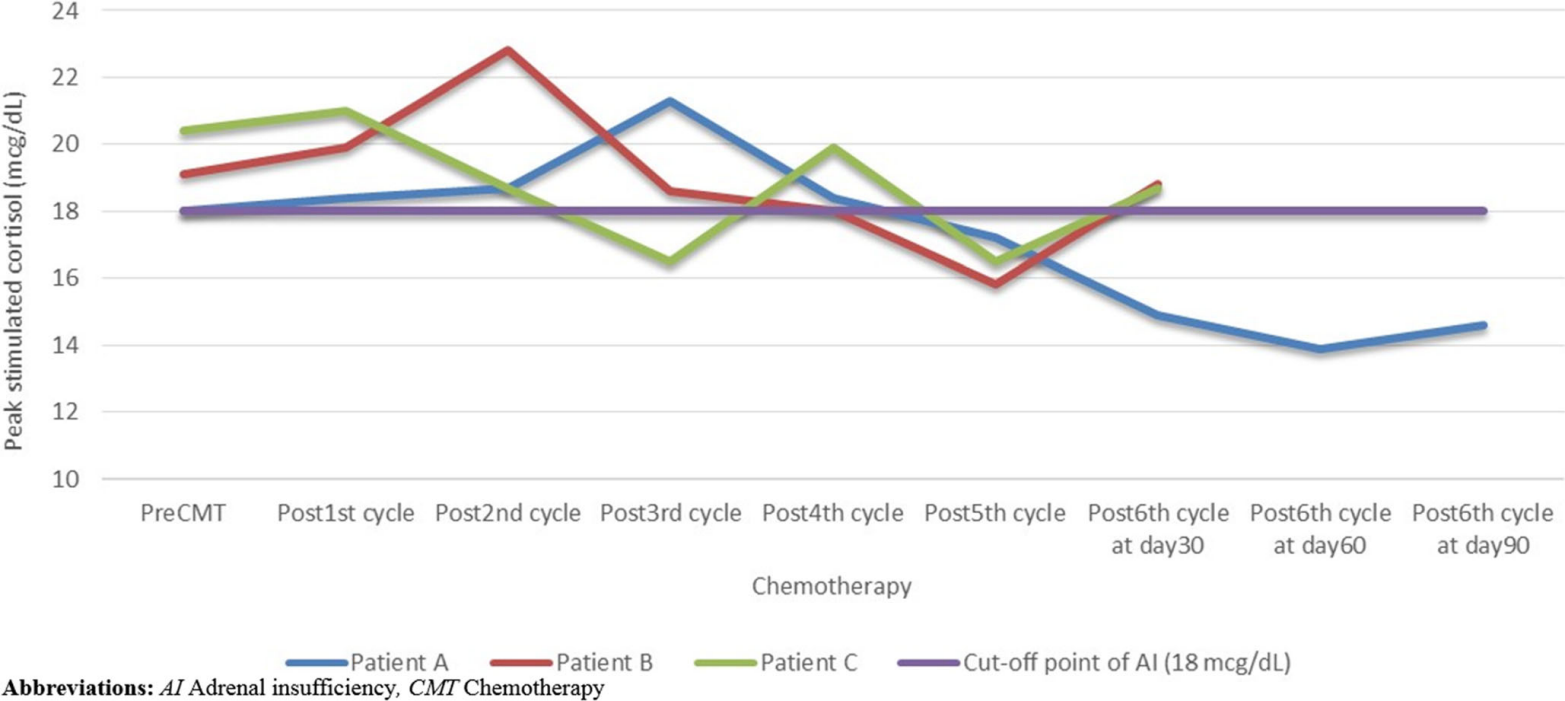
The peak-stimulated cortisol levels in adrenal insufficiency patients

The adrenal function recovered at 30 days after the last cycle of chemotherapy except in the case of 1 patient, whose HPA axis suppression persisted even after 90 days from the last chemotherapy administration. However, we observed a trend of a gradual increase in the peak-stimulated cortisol levels in this patient (Fig. 4).

In order to determine the parameters that were predictive of AI, we performed a receiver operating characteristic (ROC) analysis of the basal cortisol levels, ACTH levels, and ACTH/basal cortisol ratio from the pooled data of all 10 patients and visits. Sensitivity was calculated with those patients and time points, where the response to the 1-μg ACTH stimulation test was inadequate (5/84), and the specificity was calculated with those patients and time points, where it was normal (79/84). An adequate response to the test was defined as a peak-activated serum cortisol level above or equal to 18 μg/dL. The area under the ROC curve (AUC) to predict adrenal insufficiency showed that the AUC for basal cortisol of < 8.7 μg/dL was 0.77 (95% CI 0.56–0.98), whereas those of ACTH ≥ 26.4 pg/mL and ACTH/cortisol ratio ≥ 3.3 were 0.63 (95% CI 0.39–0.88) and 0.75 (95% CI 0.55–0.96), respectively (Table 3).

**Table 3:** Sensitivity, specificity, percentage of correctly classified patients, and positive and negative likelihood ratios of each hormonal lab finding

Lab findings	Cut-off value	AUC	Sensitivity	Specificity	Correctly classified	LR+	LR*−*
Basal cortisol	< 8.7 μg/dL	0.77 (0.56–0.98)	80%	72.2%	72.6%	2.8	0.3
ACTH	≥ 26.4 pg/mL	0.63 (0.39–0.88)	80%	50.6%	52.4%	1.6	0.4
ACTH/basal cortisol	≥ 3.3	0.75 (0.55–0.96)	80%	65.4%	66.3%	2.3	0.3

*LR+* positive likelihood ratio, *LR−* negative likelihood ratio

## Discussion

Adrenal insufficiency is a condition in which the adrenal glands fail to produce adequate amounts of cortisol. The manifestation of AI varies from being asymptomatic to an adrenal crisis, a life-threatening condition. Chronic exogenous steroid use is the most common and well-known cause of secondary AI, and AI is anticipated in patients who are exposed to an equivalent dose of more than 30 mg/day of hydrocortisone or 7.5 mg/day of prednisolone for longer than 3 weeks [12, 13].

Questions remain with regard to the occurrence of AI in patients who receive high-dose and relatively short-term corticosteroids. Those regimens are commonly used for the treatment of various hematologic malignancies, such as ALL and DLBCL. Based on previous literature, most of the data were derived from the pediatric population when high-dose systemic corticosteroids were administered during the induction period of chemotherapy for ALL. The incidence of AI ranged between 20 and 100%, varying from study to study, depending on the test methods and the cut-off cortisol values used to define an inadequate adrenal response [3]. The duration of the HPA axis recovery also ranged widely from less than 9 days to > 20 weeks. Vestergaard et al. reported a series of 96 pediatric B cell or T cell ALL patients who each received a 4-week induction combination chemotherapy, including 60 mg/m^2^/day of prednisolone administered on days 1–36, which was then tapered completely in 9 days. A 250-μg ACTH stimulation test was performed to evaluate the adrenal function within 4–6 months of discontinuation of the glucocorticoid induction. Significantly for such a population, 67% of the patients were found to have an insufficient adrenal response (i.e., a stimulated cortisol of less than 18 μg/dL at 60 min post cosyntropin administration). In this study, repeated ACTH stimulation tests were performed at 6-monthly intervals in patients with an inadequate response in order to determine the duration of the HPA axis recovery. The recovery durations were reported according to the basal cortisol levels of the first 250-μg ACTH stimulation test, which were 8.1, 5.5, and 14.7 months in patients who had a first-test basal cortisol level of < 3.62 μg/dL (< 100 nM/L), between 3.62 and 7.23 μg/dL (100–200 nM/L), and > 7.23 μg/dL (> 200 nM/L), respectively [3]. Low basal cortisol, a low delta cortisol response, and aged between 2 and 7 were associated with the insufficient adrenal response.

In another study with a comparable dosage of glucocorticoids, pediatric ALL patients received prednisolone at 60 mg/m^2^/day on days 1–7, and they were then randomized to receive either dexamethasone (10 mg/m^2^/day) or prednisolone (60 mg/m^2^/day) on days 8–29, followed by tapering of the glucocorticoids to a complete withdrawal in 9 days (day 38). In each patient, a 1-μg ACTH stimulation test (24 h after the last glucocorticoid dose) was performed on day 39. An impaired adrenal response was defined as a stimulated cortisol level of < 18 μg/dL. Patients with an insufficient response were scheduled for a repeat 1-μg ACTH stimulation test within 7–14 days and then every 2 weeks until the normal adrenal response returned. In this study, the first 1-μg ATCH stimulation test revealed an impaired adrenal response in 52/64 (81.5%) of the patients. The basal cortisol levels were < 3 μg in 35/40 (87.5%) patients, and all of them had an impaired response to the 1-μg ACTH stimulation test. Following the second ACTH stimulation 1–2 weeks later, 8/52 (15.4%) patients had AI. The adrenal function completely recovered within 10 weeks. No significant difference in the hypothalamus-pituitary-adrenal suppression or the time to adrenal recovery was found between the two groups of different steroids [1].

Our study was performed on adult patients with DLBCL; they received multiple rounds of high-dose glucocorticoids, but the duration of their exposure was shorter than the above-mentioned pediatric ALL studies. Three of our 10 patients (30%) had an inadequate adrenal response to the low-dose ACTH stimulation test. The incidence of AI in our study is lower than that reported in other studies, despite the use of a comparably high dosage of glucocorticoids per day. We hypothesize that the lower frequency of AI in our study could be due to the shorter period of exposure to steroids coupled with the 16-day steroid-free period after each CMT cycle, which provided sufficient time for the HPA axis to recover.

Interestingly, none of our patients who had an impaired adrenal response had symptoms or signs of AI, whereas in the ALL study, 18/52 patients (34.6%) had symptoms or signs of AI, such as hypotension, vomiting, anorexia, lethargy, and fever [1]. For unknown reasons, the positive cases were at a younger age and had a lower tumor burden, which contrasts with the notion that high-risk factors and an older age predispose patients to AI [10, 14–16]. The reason why our patients did not report AI-related symptoms could simply be due to the mild severity of the AI, as demonstrated by the peak-stimulated cortisol levels of close to 18 μg/dL in the AI group. For some of the more quantitative symptoms of AI, like blood pressure, this is easily explained. On the other hand, it must be kept in mind that our patients were undergoing a fairly high-dose cytoreduction therapy, which potentially leads to the masking of AI symptoms by other symptoms arising from the therapy. Thus, we feel that AI also concomitantly occurs with the use of the high-dose steroids, and patients should be monitored carefully for signs of AI. It is extremely problematic to correctly determine whether AI is present unless there are highly suspicious symptoms or signs such as hypotension, hypoglycemia, or unexplained hyponatremia. If in doubt or clinical presentations are equivocal, ACTH stimulation should be performed without delay.

The recovery period of the HPA axis observed in our study was between 3 and 5 weeks, which was shorter than in the ALL and AECOPD studies [1–3]. Possible explanations include the lower cumulative dosage and the shorter duration of the steroid exposure.

The diagnostic performance revealed by the 1-μg ACTH stimulation test for the three different cutoffs is summarized at Table 3. By way of an overall analysis for the prediction of AI, the cutoff basal cortisol concentrations < 8.7 μg/dL had high sensitivity and specificity, resulting in a likelihood ratio (LR) of 2.8 for a positive test and of 0.3 for a negative test. The results of the study provide an interesting insight: if patients receiving the CHOP regimen continue to have a basal cortisol level of < 8.7 μg/dL after the next cycle of CHOP, then AI should be suspected. A clinical assessment and biochemical testing should be conducted to confirm the diagnosis.

The strength of our study is that, to our knowledge, it is the first to demonstrate a dynamic change of the HPA axis during the whole course of chemotherapy in DLBCL patients. We have shown that HPA suppression definitely occurs in this group of patients, who receive a relatively short-term steroid therapy. This information is of value to physicians who take care of lymphoma patients who are treated with an R-CHOP/CHOP regimen. We would like to emphasize that if patients experience symptoms or signs of AI during the course of chemotherapy or several months thereafter, an immediate assessment of the HPA axis should be performed, and glucocorticoid replacement should be administered without delay in cases of infection, surgical procedures, or other stress inducers.

The study’s limitations are, firstly, its small number of recruited patients, and secondly, the fact that the pathophysiology of the higher incidence of AI in younger patients and in an earlier stage of DLBCL remains unknown. Consequently, we plan to increase the number of recruited cases in a future study to better determine the incidence of AI and, more importantly, to assess the duration of the HPA axis recovery.

## Conclusions

Transient HPA axis suppression can occur in DLBCL patients receiving short-course, high-dose corticosteroids as part of a CMT regimen. We strongly encourage careful observation and examination for potential adrenal insufficiency in these patients, particularly after the fifth cycle of chemotherapy.
